# The Emerging Oilseed Crop *Sesamum indicum* Enters the “Omics” Era

**DOI:** 10.3389/fpls.2017.01154

**Published:** 2017-06-30

**Authors:** Komivi Dossa, Diaga Diouf, Linhai Wang, Xin Wei, Yanxin Zhang, Mareme Niang, Daniel Fonceka, Jingyin Yu, Marie A. Mmadi, Louis W. Yehouessi, Boshou Liao, Xiurong Zhang, Ndiaga Cisse

**Affiliations:** ^1^Centre d’Etudes Régional Pour l’Amélioration de l’Adaptation à la SécheresseThiès, Sénégal; ^2^Laboratoire Campus de Biotechnologies Végétales, Département de Biologie Végétale, Faculté des Sciences et Techniques, Université Cheikh Anta DiopDakar, Sénégal; ^3^Key Laboratory of Biology and Genetic Improvement of Oil Crops, Oil Crops Research Institute of the Chinese Academy of Agricultural Sciences, Ministry of AgricultureWuhan, China; ^4^Centre de Coopération Internationale en Recherche Agronomique Pour le Développement, UMR AGAPMontpellier, France

**Keywords:** *Sesamum indicum*, Omic resources, molecular breeding, large-scale re-sequencing, improvement

## Abstract

Sesame (*Sesamum indicum* L.) is one of the oldest oilseed crops widely grown in Africa and Asia for its high-quality nutritional seeds. It is well adapted to harsh environments and constitutes an alternative cash crop for smallholders in developing countries. Despite its economic and nutritional importance, sesame is considered as an orphan crop because it has received very little attention from science. As a consequence, it lags behind the other major oil crops as far as genetic improvement is concerned. In recent years, the scenario has considerably changed with the decoding of the sesame nuclear genome leading to the development of various genomic resources including molecular markers, comprehensive genetic maps, high-quality transcriptome assemblies, web-based functional databases and diverse daft genome sequences. The availability of these tools in association with the discovery of candidate genes and quantitative trait locis for key agronomic traits including high oil content and quality, waterlogging and drought tolerance, disease resistance, cytoplasmic male sterility, high yield, pave the way to the development of some new strategies for sesame genetic improvement. As a result, sesame has graduated from an “orphan crop” to a “genomic resource-rich crop.” With the limited research teams working on sesame worldwide, more synergic efforts are needed to integrate these resources in sesame breeding for productivity upsurge, ensuring food security and improved livelihood in developing countries. This review retraces the evolution of sesame research by highlighting the recent advances in the “Omics” area and also critically discusses the future prospects for a further genetic improvement and a better expansion of this crop.

## Introduction

**FIGURE 1:**
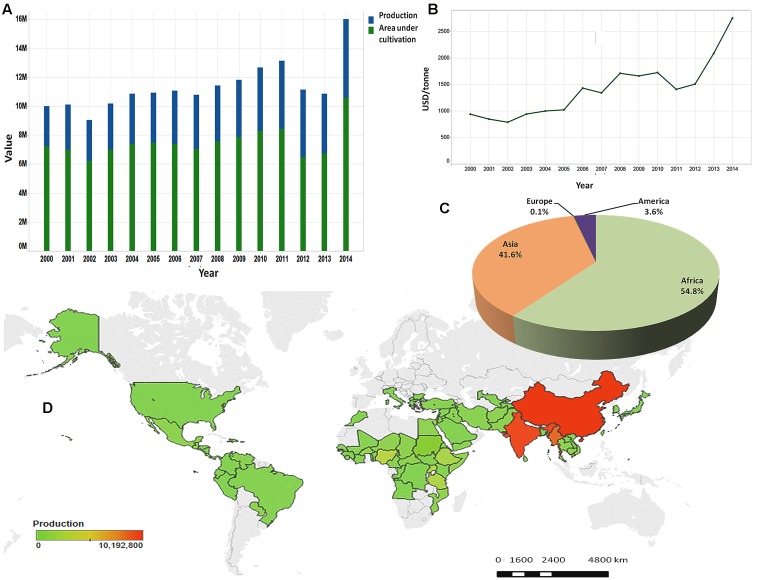
Sesame seed production in the world. **(A)** Evolution of sesame seed production and area under cultivation from 2000 to 2014. **(B)** Evolution of sale prices of sesame seed from 2000 to 2014. **(C)** Production share of sesame seed by continent in 2014. **(D)** Map of production quantities of sesame seed by country based on cumulative data from 2000 to 2014. (Source: Food and Agriculture Organization Statistical Databases [FAOSTAT], 2015).

Since the beginning of agriculture, humans have been selecting and cultivating crops that would serve their taste, energy, and health requirements (Nagaraj, 2009). Oilseeds are crops in which energy is stored mainly in the form of oil and are a very important component of semi-tropical and tropical agriculture, providing easily available and highly nutritious human and animal food (Weiss, 2000). Among the important oilseed crops widely grown in the world such as rapeseed, peanut, soybean, sunflower, sesame (*Sesamum indicum* L.) provides one of the highest and richest edible oils (Pathak et al., 2014). Sesame is a diploid species (2*n* = 2*x* = 26), an annual plant principally grown for its seeds. The seed contains 50–60% oil which has an excellent stability due to the presence of natural antioxidants such as sesamolin, sesamin, and sesamol (Anilakumar et al., 2010). The chemical composition of sesame oil characterized by a low level of saturated fatty acids (SFAs) (less than 15%) and the presence of antioxidants has been reported to have health promoting effects such as lowering cholesterol levels and hypertension in humans (Noguchi et al., 2001; Sankar et al., 2005), neuroprotective effects against hypoxia or brain damage (Cheng et al., 2006) and reducing the incidence of certain cancers (Hibasami et al., 2000; Miyahara et al., 2001). With increasing knowledge on the dietary and health benefits of sesame, the market demand for its seed and oil has enlisted a continuous steep increase. Likewise, sesame by virtue of low irrigation requirement, adaption to different types of soil and weather conditions, not being labor intensive and being instead a highly remunerative crop, is ideally suited to replace low-yield crops, especially in the current scenario of global warming affecting crop productivity in more and more traditional agricultural areas. As a result, sesame production is rapidly increasing over the years and is becoming an alternative important cash crop for smallholders, thus helping to alleviate rural poverty (**Figure 1**). In 2014, more than 6 million tons of sesame seeds have been produced under nearly 11 million ha classifying sesame at the ninth rank among the major oil crops (Food and Agriculture Organization Statistical Databases [FAOSTAT], 2015).

Despite its importance, sesame is considered as an orphan crop because it has received very little support from science, industry and policy makers. As a consequence, it lags behind the other major oilseed crops as concerns genetic improvement (Dossa, 2016). Cultivated sesame still has some wild characters including seed shattering, indeterminate growth habit and asynchronous capsule ripening leading to a very weak seed yield (300–400 Kg/ha) (Islam et al., 2016). Furthermore, sesame is often grown in harsh environments and exposed to various biotic and abiotic stresses that heavily impair its productivity (Witcombe et al., 2007). Hence, it has become crucial to enhance sesame germplasms for higher productivity and seed quality to efficiently cope with the growing demand of its oil.

Limited progress has been made in these directions through conventional breeding methods due to a lack of genomic tools and resources for deep insights into the underlying molecular background of the important agronomic traits. In addition, few scientific groups are engaged in sesame research worldwide resulting in a slow pace of sesame improvement strategies. However, in recent years, significant breakthroughs in the “Omics” area have taken sesame research into another higher stage. This has then thus propelled us to review the notable achievements made so far in this field as well as the future perspectives to speed-up sesame improvement.

## Evolutionary History of Sesame Research

**FIGURE 2:**
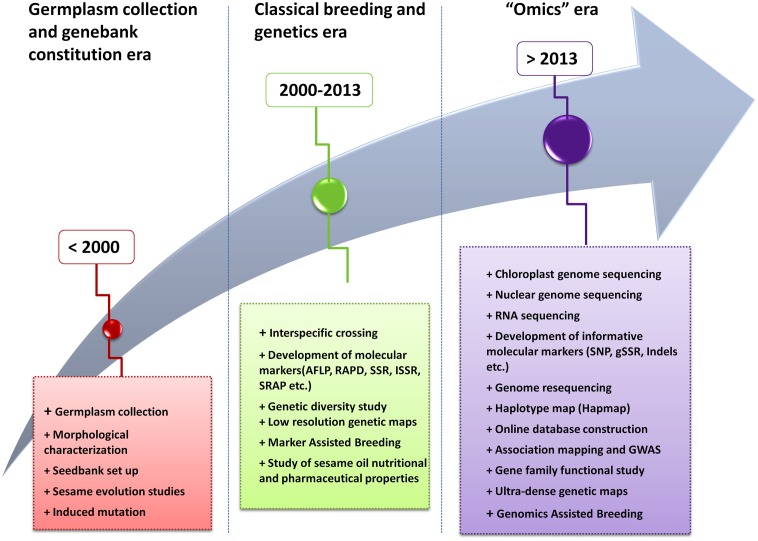
Evolutionary history of the scientific research on sesame.

Sesame is a very ancient crop thought to be one of the oldest oil crops known by humankind (Bedigian and Harlan, 1986; Ashri, 1998). Its research history followed three major periods viz. the “germplasm collection and genebank constitution” era, the “classical breeding and genetics” era, and currently the “Omics” era (**Figure 2**). During the first era (before year 2000), genetic materials of cultivated sesame as well as wild related species were collected from many growing areas, morphologically characterized and different seedbanks have been set up in several countries (Hiltebrandt, 1932; Kinman and Martin, 1954; Bedigian and Harlan, 1986; Bisht et al., 1998). Meanwhile during that period, questions related to the origin and domestication process of the cultivated sesame were the source of long debate and investigations (Hiltebrandt, 1932; Nayar and Mehra, 1970).

The second era in sesame research (2000–2013) was characterized first, by the employment of classical breeding methods including induced mutation and screening of genotype for desirable characters (Wongyai et al., 2001; Uzun et al., 2003; Boureima et al., 2012). Afterward, sesame research has witnessed a rapid development of genetic tools particularly molecular markers and their application in genetic diversity studies and marker assisted breeding (Dixit et al., 2005; Wang et al., 2012b; Wei X. et al., 2014). In addition, during that period of time, several studies have been undertaken on sesame oil properties that shed more light on the nutritional, pharmaceutical and engineering applications of this untapped crop (Miyahara et al., 2001; Cheng et al., 2006; Saydut et al., 2008; Anilakumar et al., 2010).

Finally, since 2013, the sesame research enters the “Omics” era. With the completion of the nuclear and chloroplast genome sequencing as well as the release of various transcriptomic data, enormous genomic resources have been generated and being applied for sesame improvement.

## Genetic Resources

The *Sesamum* genus belongs to the Eudicotyledon clade, Lamiales order, Pedaliaceae family (Mabberley, 1997) and *S. indicum* is the well-known and widely grown species within this genus (Ashri, 1998). Kobayashi et al. (1990) proposed 36 species belonging to this genus including 22 species found exclusively in the African continent, five in Asia, seven commonly found in Africa and Asia and one species each in Brazil and a Greek island. Later on, based on works of Bedigian, the list of *Sesamum* species has been revisited to 23 species (IPGRI and NBPGR, 2004) (**Table 1**). Beside *S. indicum*, the species *S. radiatum* is also cultivated in some African countries as leafy vegetables.

**Table 1:** Revised list of *Sesamum* species and their chromosomes number (2*n*).

2*n* = 26	2*n* = 32	2*n* = 64	2*n* = indeterminate
*S. alatum* Thonn.	*S. capense* Burm.f. ssp. lepidotum Schinz	*S. radiatum* Schum. & Thonn.	*S. abbreviatum* Merxm.
*S. capense* Burm.f.	*S. angolense* Welw.		*S. calycinum* Welw. ssp. calycinum
*S. indicum* L.	*S. angustifolium* Engl.		*S. calycinum* Welw. ssp. baumii (Stapf) Seidenst. ex. Ihlenf
*S. malabaricum* Burm.	*S. laciniatum* Wild.		*S. calycinum* Welw.ssp. pseudoangolense Seidenst ex.Ihlent
	*S. latifolium* Gillet		*S. marlothii* Engl.
	*S. prostratum* Retz.		*S. parviflorum* Grabow-Seidenst
			*S. pedalioides* Heirn
			*S. rigidum* Peyr. ssp. rigidum
			*S. rigidum* ssp. merenksyanum Ihlenf. & Seidenst
			*S. schinzianum* Aschers. ex. Schinz
			*S. triphyllum* Welw. ex. Aschers
			*S. triphyllum* Welw. ex. Aschers. var. *grandiflorum* (Schinz) Merxm


*Source: IPGRI and NBPGR, 2004.*

Because most of wild species of the *Sesamum* genus exist only in Africa, sesame has been thought to be originated from this continent (Hiltebrandt, 1932). However, according to evidence in studies Bedigian’ (2003, 2004), it is assumed that the crop has been domesticated from its wild relative species *S. malabaricum* native to south Asia and spread west to Mesopotamia before 2000 B.C. (Fuller, 2003). Sesame harbors a huge diversity probably because of an adaptation to the various environments where its presence has been recorded coupled with long-term natural and artificial selections (Bedigian and Harlan, 1986; Wei et al., 2015). In total, five major centers of diversity have been proposed for sesame including India, China, Central Asia, the Middle-East and Ethiopia (Zeven and Zhukovsky, 1975). Thanks to the meaningful efforts of the scientific community in sesame germplasm collection, characterization and conservation, huge genetic materials of cultivated sesame along with wild related species are currently preserved in several genebanks around the world mainly in Asia (Zhang Y. et al., 2012) (**Table 2**). The principal genebanks of sesame held in India (NBPGR National Gene Bank), in South Korea (National Agrobiodiversity Center, Rural Development Administration; Park et al., 2015), in China (Oil Crops Research Institute, Chinese Academy of Agricultural Sciences; Wei et al., 2015) and in United States (USDA, ARS, PGRU), have preserved about 25,000 genetic materials (**Table 2**). Moreover, several small-scale genebanks exist in some African countries including Nigeria, Ethiopia, Sudan, etc. Since these genebanks harbor important quantity of genetic resources, it is important to establish core collections (CC) which is a favored approach for the efficient exploration and utilization of novel variations in genetic resources (Hodgkin et al., 1995). In this vein, researches on sesame CC establishment have been conducted resulting in 362 accessions for Indian germplasm (Bisht et al., 1998), 453 accessions for Chinese germplasm (Zhang et al., 2000) and 278 accessions for Korean germplasm (Park et al., 2015). These are the reservoirs of genetic resources for the present and future sesame improvement programs. Unfortunately, utilization of these wealthy genetic resources for sesame improvement is very limited and most of diversity existing in the germplasm remains unexplored (Dossa et al., 2016a). Furthermore, it becomes apparent that sesame genetic resources from Asia have been well characterized and preserved in contrast to African germplasm which also harbors a valuable diversity (Dossa et al., 2016a). Therefore, further exertions are needed to gather locally available sesame accessions and wild related species from Africa and constitute an extensive genebank for their efficient conservation and exploitation.

**Table 2:** List of worldwide major genebanks available for sesame species.

Country	Institut	Accession numbers	Website
India	NBPGR National Gene Bank	∼10,000	www.nbpgr.ernet.in
South Korea	National Agrobiodiversity Center, Rural Development Administration	∼7,698	http://www.rda.go.kr/foreign/ten/
China	Oil Crops Research Institute	∼7000	http://www.sesame-bioinfo.org/phenotype/index.html
United States	USDA-ARS- PGRU	∼1,226	www.ars.usda.gov


## “Omics” Resources

The genetic and molecular biology study of sesame began very late with only one genetic map published and no report on quantitative trait loci (QTL) mapping before 2013. However, over the last few years, some significant progress has been made in the development of large-scale genomic resources including informative molecular markers, ultra-dense genetic maps, transcriptome assemblies, multi-omics online platforms etc. In addition, the release of the draft genome of sesame (Wang et al., 2014a) triggered functional analyses of candidate genes related to key agronomic traits. With these invaluable efforts, sesame holds some important genomic resources and platforms for its improvement which for the time being are inexistent in some important oilseed crops such as groundnut. Similarly like pigeonpea (Pazhamala et al., 2015), chickpea, millets (Varshney et al., 2009, 2010), sesame has graduated from an “orphan crop” to a “resource-rich crop”.

### Molecular Markers

Molecular marker technologies have significantly speeded up modern plant breeding in enhancing the genetic gain and reducing the breeding cycles in many crop species. Different types of molecular marker systems have been developed and applied to sesame genotyping and breeding efforts. The first class of molecular markers including Random Amplified Polymorphic DNA (RAPD; Bhat et al., 1999) and Amplified Fragment Length Polymorphism (AFLP; Laurentin and Karlovsky, 2006) were designed and employed mainly for genetic diversity studies. The second class of markers involved basically Simple Sequence Repeat (SSR) types such as Inter-Simple Sequence Repeats (ISSR; Kim et al., 2002), Expressed Sequence Tags-SSR (EST-SSR; Wei et al., 2008, 2011; Badri et al., 2014; Sehr et al., 2016), cDNA-SSR (Spandana et al., 2012; Wang et al., 2012b; Zhang H. et al., 2012; Surapaneni et al., 2014; Wu et al., 2014b), Genome sequence-SSR (gSSR; Dixit et al., 2005; Wei X. et al., 2014; Uncu et al., 2015; Dossa, 2016; Yu et al., 2016), Chloroplast SSR (cpSSR, Sehr et al., 2016). By compiling all developed SSR marker resources, there are in total more than 7,000 validated and 100,000 non-validated SSR markers available for sesame research. Interestingly, a new study is underway to set up an online database gathering all SSR information and providing an integrated platform for functional analyses in sesame. Many of these markers were used for genetic and association mapping, molecular breeding and genetic diversity studies in sesame (Wei et al., 2013; Li et al., 2014; Liu et al., 2015; Uncu et al., 2015; Dossa et al., 2016a). Finally, in recent years with the next generation sequencing (NGS) technology, the third class of molecular markers came into existence. SNPs are more useful as genetic markers than many conventional markers because they are the most abundant and stabile form of genetic variation in most genomes. Therefore, the available high-throughput methods for SNP discovery and genotyping have been employed in sesame research including Restriction site-Associated DNA sequencing (RAD-seq; Wu et al., 2014a; Wang et al., 2016a), Specific Length Amplified Fragment Sequencing (SLAF-seq; Zhang et al., 2013), RNA-Seq (Wei L. et al., 2014), Whole-Genome Sequencing (WGS; Wang et al., 2014b; Wei et al., 2015; Zhang et al., 2016), Genotyping by sequencing (GBS; Uncu et al., 2016). Another important marker system referred as insertion/deletions (Indels) has also been reported in sesame (Wei L. et al., 2014; Wu et al., 2014a).

As a whole, molecular marker technologies in sesame are witnessing considerable progress and it is obvious that sesame is no longer lagging far behind major crops in this field.

### Genome Sequence Resources

A high-quality reference genome sequence provides access to the relatively complete gene catalog for a species, the regulatory elements that control their function and a framework for understanding genomic variation. As such, it is a prerequisite resource for fully understanding the role of genes in development, driving genomic-based approaches to systems biology and efficiently exploiting the natural and induced genetic diversity of an organism (Feuillet et al., 2011). Researchers from Oil Crops Research Institute of the Chinese Academy of Agricultural Sciences, BGI and other institutes have successfully cracked the nuclear genome of sesame, generating 54.5 Gb of high-quality data from the elite cultivar “Zhongzhi No.13” using the Illumina Hiseq2000 platform. This has been the major breakthrough in the sesame research for decades (Wang et al., 2014a). The high-quality draft genome encompassing 27,148 genes distributed on 16 Linkage Groups (LG) with 274 Mb of size, has become the reference genome for biology study in sesame^1^. This genome with a contig N50 of 52.2 kb and a scaffold N50 of 2.1 Mb has been recently upgraded to reach 13 pseudochromosomes, 94.3% of the estimated genome size and 97.2% of the predicted gene models in sesame (Wang et al., 2016a). In parallel, another genome sequencing project was initiated under the auspices of the Sesame Genome Working Group (SGWG). By 2013, they assembled from the variety “Yuwhi 11” a genome size of 293,7 Mb out of the 354 Mb estimated in sesame and predicted the function of 23,713 genes^2^. More recently, two new genome sequences from sesame landraces (“Baizhima” and “Mishuozhima”) have also been released, increasing the genome sequence resources available for this crop (Wei et al., 2016).

In addition, works carried out by a team of National Bureau of Plant Genetic Resources from India resulted in the genome sequencing of the Indian variety^3^ “Swetha.”. Of note, nearly 1000 sesame accessions and mapping population have been re-sequenced providing tremendousum and inestimable genome-wide information (Zhang et al., 2013a, 2016; Wang et al., 2014b, 2016a; Uncu et al., 2015, 2016; Wei et al., 2015). Nowadays, gene family study, gene fine-mapping, gene cloning and molecular breeding, genome wide association studies (GWAS), genome variation and evolution studies are feasible (Wei et al., 2015, 2016; Dossa et al., 2016b,d; Yu et al., 2017). Novel breeding approaches such as genomic selection (GS) could be implemented in sesame and accelerate the crop improvement.

Beyond the nuclear genome sequences of sesame, the chloroplast genome has also been decrypted first, in a black-seeded cultivar “Ansanggae” (Yi and Kim, 2012) and subsequently, in a white-seeded cultivar “Yuzhi11” (Zhang et al., 2013b). These studies indicated that *Sesamum* (Pedaliaceae family) is a sister genus to the *Olea* and *Jasminum* (Oleaceae family) clade and represents the core lineage of the Lamiales families.

### Transcriptome Assembly

**FIGURE 3:**
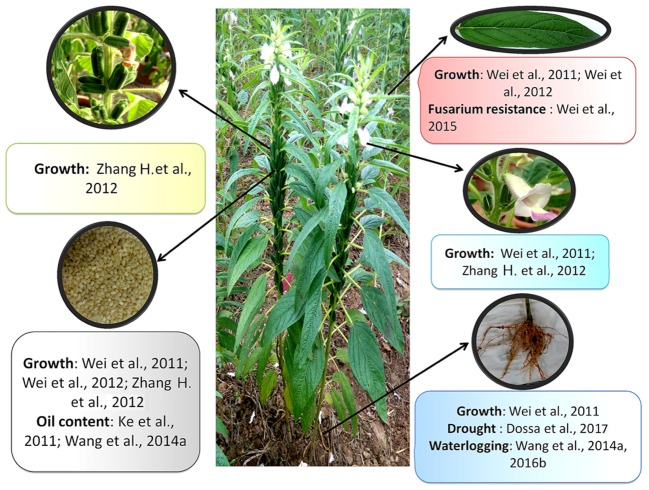
Transcriptome data available and tissues/traits investigated.

Transcriptome or EST sequencing is the first step to access the gene contents of a species and has emerged to be an efficient way to generate functional genomic data for non-model organisms. Like genome sequence resources, sesame holds several transcriptome data generated from various organs of the plant (**Figure 3**). The first transcriptome profiling began with works of Suh et al. (2003) who obtained 3,328 ESTs from a cDNA library of 5–25 days old immature sesame seeds. This study shed light on the metabolic pathways involved in lignan biosynthesis in sesame including sesamin and sesamolin. Wei et al. (2011) sequenced five tissues using for the first time the high-throughput Illumina paired-end sequencing technology. Likewise, works of Wei et al. (2012), Zhang H. et al. (2012), Wang et al. (2014a) yielded various transcriptome resources related to sesame growth and developmental stages using various sequencing technologies including Illumina HiSeq 2000 and GAII. These studies increased our understanding on the genomic background underpinning sesame growth and development.

On the other hand, given that sesame productivity is seriously hampered by different biotic and abiotic stresses, studies have been designed to unravel the molecular basis of stress tolerance and find out some potential genes to impart stress tolerance in sesame genotypes. The transcriptional response of root tissues to waterlogging stress was investigated in sesame root (Wang et al., 2012c). More recently based on the Illumina 2500 platform, a time-course transcriptome profiling in two sesame genotypes displaying contrasting tolerance levels provided substantial gene expression data for sesame responses to waterlogging stress (Wang et al., 2016b). Another important abiotic stress impairing sesame productivity is drought stress which is still poorly characterized at the molecular level (Dossa et al., 2016b). For this purpose, Dossa et al. (2017) explored gene expression changes in two sesame genotypes (tolerant and sensitive) based on Illumina HiSeq 4000 sequencing platform, under progressive drought and after re-watering so as to find out some potential genes associated with drought tolerance. Currently, efforts are underway to decipher sesame molecular responses to salt stress, another important abiotic stress.

Concerning the biotic stress very little research has been undertaken at the molecular level, urging more investigations for the development of disease resistant crop. RNA-seq study has been performed on resistant and susceptible sesame cultivars inoculated with *Fusarium oxysporum* f. sp. sesami to clarify the molecular mechanism of sesame resistance to Fusarium Wilt which is one of the main worldwide diseases in sesame, resulting in 15–30% losses of yield (Li et al., 2012; Wei et al., 2016).

Finally, transcriptome profiling approach has also been deployed to explore other important traits. For example, Liu et al. (2016) compared two near-isogenic lines [W1098A with dominant genic male sterility (DGMS) characteristic and its fertile counterpart, W1098B] to identify differentially expressed genes related to male sterility.

To sum up, huge transcriptome data based on state-of-the-art sequencing technologies from various tissue samples and experimental conditions are now available in sesame and have increased our knowledge on sesame biology. These resources could assist in upgrading the current reference genome in the near future. In this way, a functional transcriptome database should be built to facilitate the exploitation of these incommensurable resources.

### Development of Genetic Maps and Breeding Populations

A genetic linkage map is a prerequisite to better understand the inheritance of traits at the genome-wide level (Verma et al., 2015). It helps to identify molecular markers associated with relevant traits that can be used in breeding programs. The first genetic map in sesame was constructed in 2009 using a combination of 220 EST-SSR, AFLP and Random Selective Amplification of Microsatellite Polymorphic Loci (RSAMPL) markers (Wei et al., 2009). It was built from a F_2_ population COI1134 × RXBS and encompassed 30 LGs covering a genetic length of 936.72 cM with an average marker interval of 4.93 cM (**Table 3**). At that period, the number of informative molecular markers was limited hence, the development of linkage maps with a good resolution was challenging. Subsequently, this map has been improved to obtain 14 LGs spanning a genome distance of 1,216 cM. In total, 653 markers were successfully mapped with a marker density of 1.86 cM per marker interval. Thanks to the good resolution reached in this linkage map, QTL identification for seed coat color has been conducted for the first time in sesame (Zhang et al., 2013c). During the same period, another linkage map was released based on Specific-Locus Amplified Fragment sequencing (SLAF-seq) technology (Zhang et al., 2013). This map based on an F_2_ population Shandong Jiaxiang Sesame × Zhongzhi No.13, comprised 1,233 SLAF markers that are distributed on 15 LGs, and was 1,474.87 cM in length with average marker spacing of 1.20 cM. The density of this map was higher than the previous ones and it was the first linkage map to integrate SNP markers in sesame. Surprisingly, all these three published linkage maps lacked common markers. Furthermore, an important knot is that they were constructed from temporary population (F_2_) that renders repeated phenotyping unfeasible, hence were not ideal for quantitative traits mapping. In this regard, another high-throughput sequencing technology named RAD-seq, has been adopted by Wu et al. (2014a) to map in a high-density linkage map nearly 1,230 markers and identified several QTLs linked to grain yield-related traits based on a recombinant inbred line (RIL) population from Zhongzhi14 × Miaoqianzhima.

**Table 3:** Genetic mapping and association mapping studies in sesame.

Authors	Traits	Marker systems	Marker number	Population	Major results
Wei et al., 2009	–	EST-SSR, AFLP and RSAMPL	220	96 F_2_	First genetic map
Zhang et al., 2013b	Seed coat color	SSR, AFLP and RSAMPL	653	BC_1_, BC_2_, F_2_	4 QTLs
Zhang et al., 2013c	–	SLAFseq	1,223	107F_2_	First dense genetic map
Wei et al., 2013	Seed quality traits	SRAP, SSR and AFLP	79	216 Chinese collection	10 markers associated with oil, oleic acid, linoleic acid and protein
Wu et al., 2014a	Grain yield traits	SNP	1,230	224 RIL	30 QTLs
Li et al., 2014	Oil and protein content	SSR	112	369 worldwide accessions	19 SSR linked to oil content and 24 SSR associated with protein content
Wei et al., 2015	56 agronomic traits	SNP	1,800,000	705 worldwide accessions	549 associated loci and 46 causative genes linked to key agronomic traits
Wang et al., 2016a	Plant height and seed coat color	SNP	1,522 bins	430 RIL	41 QTLs linked to plant height and 9 QTLs linked to First genetic map with 13 LGs
Wei et al., 2016	Seed coat color	SSR	400	550 F_6_	6 QTLs linked to seed coat color and Identification of *PPO* gene related to black seed coat color
Zhang et al., 2016	Determinate growth habit	SNP	30,193	120 F_2_	First ultra-dense genetic map with 13 LGs Identification of the determinacy gene *SiDt*
Uncu et al., 2016	–	SNP and SSR	432	93 RIL	Genetic map with 13 LGs
Mei et al., 2017	Number of flowers per axil and Branching habit	SLAF	9,378	150 BC_1_	High-density genetic map with 13 LGs, identification of *SiFA* and *SiBH* linked to Number of flowers per axil and Branching habit, respectively


Zhang et al. (2014) also developed a RIL population from Zhongzhi No.13 × Yiyangbai to analyzed QTLs related to waterlogging tolerance. Furthermore, linkage analysis strategy has been applied in an inter-specific population Ezhi1 (*S. indicum* L., 2*n* = 26) × Yezhi2 (*S. mulayanum* Nair, 2*n* = 26) and a RIL from 95 ms-5A × 95 ms-5B to study genic male sterility (GMS) traits (Zhao et al., 2013; Liu et al., 2015).

Although the resolution in sesame genetic mapping steeply increased over the years, none of the published maps included the same number of LGs as the number of chromosomes known in sesame. It was just recently that the significant works of Wang et al. (2016a), Zhang et al. (2016), and Mei et al. (2017) have permitted to reach this milestone. The first map was based on 430 RILs from Zhongzhi No.13 × ZZM2748 (semi-dwarf) and included 1,522 bins anchored on 13 LGs spanning 1090.99 cM genome length with a mean interval distance of 0.72 cM between adjacent bins. This bin map was used to identify several QTLs for sesame plant height and seed coat color (Wang et al., 2016a). The second one was constructed on the basis of a F_2_ population from Yuzhi11 (indeterminate growth) × Yuzhi DS899 (determinate growth). This SNP map was comprised of 3,041 bins including 30,193 SNPs in 13 LGs with an average marker density of 0.10 cM. At present, this is densest linkage map in sesame and was efficiently applied for map-based gene identification (Zhang et al., 2016). The third one was built on 150 BC_1_ from Yuzhi4 × Bengal Small-seed and encompassed 9,378 SLAF markers anchored onto 13 LGs spanning a total genetic distance of 1,974.23 cM with an average genetic distance of 0.22 cM.

Worth noting, a recent linkage map has been constructed from a RIL population Acc. No. 95–223 × Acc. No. 92–3091 and yielded 13 LGs encompassing 914 cM with 432 markers including 420 SNPs and 12 SSRs (Uncu et al., 2016). Besides, a haplotype map (Hapmap) has been constructed from 705 worldwide accessions providing 5,407,981 SNPs information with an average LD decay rate of 88 kb in the whole sesame genome. This genotyped worldwide panel has been selected as a “training population” which constitutes a tremendous resource for GWAS, Genomic Selection (GS) and evolution studies in sesame (Wei et al., 2015).

Definitively, from the first low-resolution genetic map constructed in 2009 to the ultra-dense and high-resolution recent ones, there is evidence that the genetic research in sesame has impressively improved and has reached a new dimension. Nonetheless, in order to use these map resources to their maximum advantage, it would be ideal to construct a consensus map that will provide a framework of unprecedented marker density and genome coverage for fine QTL analysis, association mapping, thus facilitating the application of molecular breeding strategies in diverse sesame germplasms. In this regard, an effective collaboration between the different sesame working teams will help achieve this goal.

### Online Functional Database Resources

Owing to the multitude genomic sequence resources being released for sesame research, several integrative online databases have been created to gather sesame information and providing user-friendly platforms for researchers to easily study the molecular function of genome components, for comparative genomics and breeding applications (**Table 4**). The first web-based functional platform on sesame was set by the Sesame Genome Project but it does not supply detailed sesame genomic or genetic data at the time. Hence, after the release of the reference genome, a versatile online database referred to as Sinbase^4^ was developed and provides digestible information related to sesame genomics and genetics (Wang et al., 2014c). This database included genomic component annotations to allow users to study sesame more thoroughly; genetic linkage groups for gene cloning; QTL for genetic linkage analyses; colinear blocks and orthologous genes to perform comparative and evolutionary analyses in sesame and other related species. Additionally, extensive phenotyping data were supplied describing variations in sesame plant (growth habits, branching styles, number of flowers and capsules per axil, number of carpels in a capsule, flower colors, capsule length etc.). Overall, 10 comprehensive online databases are currently available to study sesame biology including five platforms focusing solely in sesame on aspects related to genome functional components, gene expression, SSR, SNP, Indels, Transposons, QTL and functional genes, gene family, comparative genomics, genetic maps, haplotype map, phenotypes, etc. (Zhang et al., 2013a; Wang et al., 2014c; Ke et al., 2015; Yu et al., 2015, 2016; Wei et al., 2016, 2017).

**Table 4:** List of available online databases for functional genomics in sesame.

Database name	Website	Utility	Reference
Sinbase	http://www.ocri-genomics.org/Sinbase/index.html	Genomics/Comparative genomics/Genetics/Phenotypes etc.	Wang et al., 2014c
SesameHapMap	http://202.127.18.228/SesameHapMap/	Genome wide SNP	Wei et al., 2015
SesameFG	http://www.ncgr.ac.cn/SesameFG	Genomics/Evolution/breeding/comparative genomics/Molecular markers/Phenotypes/Transcriptomics	Wei et al., 2017
SisatBase	http://www.sesame-bioinfo.org/SisatBase/	Genome wide SSR	–
The Sesame Genome Project	http://www.sesamegenome.org	Genomics	Zhang et al., 2013
Sesame Germplasm Resource Information Database	http://www.sesame-bioinfo.org/phenotype/index.html	Plant phenotype	–
NCBI^∗^	http://www.ncbi.nlm.nih.gov/genome/?term=sesame	Versatile	–
ocsESTdb^∗^	http://www.ocri-genomics.org/ocsESTdb/index.html	Seed expression sequence tags/comparative genomics	Ke et al., 2015
PTGBase^∗^	http://www.ocri-genomics.org/PTGBase/index.html	Tandem duplication/evolution	Yu et al., 2015
PMDBase^∗^	http://www.sesame-bioinfo.org/PMDBase	SSR information	Yu et al., 2016


*^∗^These database involved several species including sesame.*

## Application of Newly Developed “Omics” Tools in Sesame Research

**FIGURE 4:**
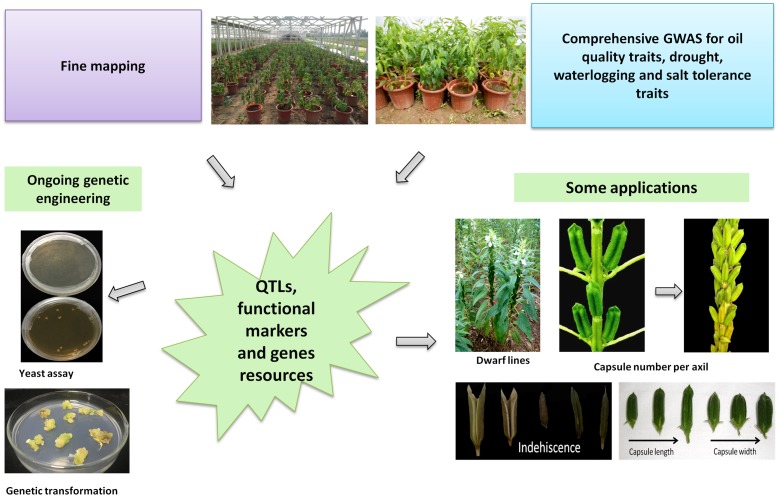
Deployment of newly developed “Omics” tools in sesame improvement strategies.

With mounting development of versatile “Omics” tools in sesame, their application and deployment for the crop improvement strategies have also yielded conspicuous results. Nowadays, these tools enable high efficiency and resolution in genetic diversity study, gene-trait association analysis using bi-parental or natural diverse populations, gene family study, RNA-seq based candidate gene identification. Various traits of the plant have been tagged by researchers including oil content and quality traits, yield components, tolerance to drought and waterlogging stresses, disease resistance, good plant architecture etc. A glimpse of the diverse applications of “Omics” tools in sesame research is presented in **Figure 4**.

### Germplasm Characterization

The availability of molecular markers has paved the way for several genetic diversity studies in sesame. Knowledge on the genetic diversity and population structure of germplasm collections is an important foundation for crop improvement and a key component of effective conservation and breeding strategies (Thomson et al., 2007). Pathak et al. (2014) have previously reviewed in detail the numerous genetic diversity studies conducted on sesame germplasms. It emerges that sesame harbors a valuable diversity and incongruence between geographical proximity and genetic distance has been reported (Bhat et al., 1999; Laurentin and Karlovsky, 2006; Abdellatef et al., 2008; Pham et al., 2009; Zhang Y. et al., 2012; Alemu et al., 2013; Abate et al., 2015). However, few informative and polymorphic markers were used in these studies and might not accurately distinguish the samples. Since the genome sequence of sesame becomes available, thousands of highly informative markers including gSSRs, SNPs and Indels covering the entire genome, have been developed and applied for genetic diversity studies (Wang et al., 2014b; Wei X. et al., 2014; Wu et al., 2014b; Wei et al., 2015; Uncu et al., 2015; Dossa et al., 2016a). These studies in contrast, have proved certain patterns of association between genetic similarity and geographical proximity in sesame. Even though the exchange of sesame materials between diverse locations especially through seed trading increases gene flow, in most of cases, some locally unique gene pools still exist. For example, Dossa et al. (2016a) investigated for the first time the unknown sesame accessions from West Africa and found that they were unique and distinct from all the rest. These observations suggested that newly developed molecular markers would bring more precision and efficiency in both genetic studies and breeding programs. Furthermore, combination of molecular and morphological characterizations has been employed to assess the diversity in sesame germplasm. A total of 137 Turkish germplasm has been characterized using both morphological and molecular data which led to the selection of a core collection useful for sesame preservation and breeding (Frary et al., 2014). Ugandan sesame landraces were also investigated and results showed incongruence between morphological data and molecular data (Sehr et al., 2016). Similarly, Pandey et al. (2015) analyzed a worldwide germplasm dominated by Indian accessions. They detected a high genetic diversity within the germplasm but there was an insignificant correlation between phenotypic and molecular marker information which highlighted the importance to associate both genetic and phenotypic diversity to efficiently inform on the extent of the variation present in sesame germplasm.

Concerning wild related species, very few genetic studies have been conducted for their characterization. Uncu et al. (2015) uncovered a high rate of marker transferability between *S. indicum* and *S. malabaricum*, supporting the designation of the two taxa as cultivated and wild forms of the same species. In earlier works, Nyongesa et al. (2013) found that, there is a high genetic diversity within wild sesame species. The clustering pattern of wild and the cultivated forms indicated that there is no cross-pollination between them during domestication. In addition, it was proved that the genetic diversity of sesame had been eroded due to selection after domestication (Wu et al., 2014b; Wei et al., 2015; Pathak et al., 2015; Mondal et al., 2016). Therefore, future sesame cultivation would benefit from the incorporation of alleles from sesame’s wild relatives. Wild species of sesame possess genes for resistance to major biotic and abiotic stresses and adaptability to different environments (Joshi, 1961). Unfortunately, in contrast to several crops such as groundnut, cotton, sunflower, rice, maize, wheat, tomato, soybean, etc., which are profiting from their wild related species for the improvement of cultivars (Zamir, 2001), the introgression of useful genes from wild species into cultivars via conventional breeding has not been so far successful in sesame mainly due to post-fertilization barriers (Tiwari et al., 2011).

### Functional Genomics Research for Key Agronomic Traits

#### Oil and Quality Traits

Sesame is primarily grown for its oil-bearing seed. Beside the high oil content, sesame seeds contain almost 18% proteins and among the fatty acid compositions, oleic acid (39.6%) and linoleic acid (46%) are the two main components with the ideal ratio of almost 1:1 (Anilakumar et al., 2010). Wherefore, numerous studies have early attempted to decipher the genetic basis of the oil yield and quality which are some key agronomic traits in sesame breeding. Even so, until 2013, the molecular mechanisms of the high oil content and quality in sesame seeds were still unclear (Jin et al., 2001; Chun et al., 2003; Suh et al., 2003; Ke et al., 2011). An association mapping of oil content, protein content, oleic acid concentration, and linoleic acid concentration based on multi-environment trials was conducted using 79 SSR, SRAP, and AFLP markers in 216 Chinese sesame accessions (Wei et al., 2013). Only one associated marker (*M15E10-3*) was identified for oil content in two environments suggesting inadequate molecular markers and/or germplasm resources. In this regard, Li et al. (2014) analyzed 369 sesame accessions with larger phenotypic variation under 5 environments using 112 informative SSRs. A total of 19 and 24 SSRs were detected for oil content and protein content, respectively. From these, 19 markers were shared by both traits suggesting that oil and protein contents are controlled mostly by similar and major genes. By combining genome information (Zhang et al., 2013a) to the association mapping results, 36 candidate genes related to lipid pathway including fatty acid elongation gene and a gene encoding Stearoyl-ACP Desaturase were identified. Later, Chen et al. (2014) investigated the sequence divergence in the coding region of the Fatty acid desaturase (FAD) gene between wild and cultivated sesame. They found some nucleotide polymorphisms located in enzyme active site between the wild and cultivated forms which may contribute to the higher fatty acid composition in the cultivated sesame. The specific primers linked to these functional SNPs would be for a great importance in molecular breeding toward high fatty acid content in sesame varieties.

The release of the reference genome sequence has provided an unparalleled opportunity to further excavate the molecular basis of high oil content and quality traits in sesame (Wang et al., 2014a). The sesame genome was found to harbor low copy of lipid-related genes (708) compared to related species such as soybean (1,298). This finding was unexpected since there is an obvious difference in oil contents between sesame (∼55%) and soybean (∼20%). More interestingly, by combining comparative genomic and transcriptomic analyses, authors have discovered that some lipid gene families, especially the transfer protein type 1 (LTP1) genes beneficial for high oil accumulation have been expanded and retained during domestication, while lipid degradation-related families were found reduced in sesame compared to soybean and this may underlay sesame high oil content. Additionally, important genes in the triacylglycerol biosynthesis pathway were found highly implicated in the oil accumulation during early stages of sesame seed development. Finally, two potential key genes *SiDIR* (*SIN_1015471*) and *SiPSS* (*SIN_1025734*) were detected for sesamin production in sesame (Wang et al., 2014a).

Genome wide association studies takes full advantage of ancient recombination events to identify the genetic loci underlying traits at a relatively high resolution (Huang and Han, 2014). In a comprehensive GWAS for oil and quality traits in 705 sesame accessions under 4 environments, 13 significant associations were unraveled for oilseed compounds including oil, protein, sesamin, sesamolin, SFA, Unsaturated Fatty Acid (USFA) and the ratio SFA/USFA. Several causative genes were uncovered for oil content [*SIN_1003248*, *SIN_1013005*, *SIN_1019167*, *SIN_1009923*, *SiPPO* (*SIN_1016759*) and *SiNST1* (SIN*_1005755*)], for fatty acid composition [*SiKASI* (*SIN_1001803*), *SiKASII* (*SIN_1024652*), *SiACNA* (*SIN_1005440*), *SiDGAT2* (*SIN_1019256*), *SiFATA* (*SIN_1024296*), *SiFATB* (*SIN_1022133*), *SiSAD* (*SIN_1008977*), *SiFAD2* (*SIN_1009785*)], for sesamin and sesamolin content [*SiNST1* (*SIN_1005755*)] and protein content [*SiPPO* (*SIN_1016759*)].

Further studies of Zhang et al. (2013), Wang et al. (2016a) resulted in 4 QTLs (*QTL1-1*, *QTL11-1*, *QTL11-2* and *QTL13-1*) and 9 QTLs (*qSCa-8.2*, *qSCb-4.1*, *qSCb-8.1*, *qSCb-11.1*, *qSCl-4.1*, *qSCl-8.1*, *qSCl-11.1*, *qSCa-4.1* and *qSCa-8.1*) detected for seed coat color, respectively. Additionally, the gene *SiPPO* (*SIN_1016759*) has been recently detected through fine mapping, as the candidate gene that controlling seed coat color in sesame (Wei et al., 2016). Seed coat color is an important agronomic trait in sesame, as it has been shown that white sesame seeds typically have higher oil, sesamin or sesamolin content (Wang et al., 2012a), whereas black sesame seeds usually have higher ash and carbohydrate content and lower protein, oil, and moisture ratios (Kanu, 2011). Though, these QTLs harbor dozen of genes, further screening will help to pinpoint the candidate genes.

Compiling all these meaningful results regarding oil and quality traits, researchers actually have substantial genomic information at their disposal for breeding and releasing higher nutritional cultivars to meet the various demands of oil markets.

#### Waterlogging and Drought Tolerance

Sesame is highly susceptible to waterlogging stress. The crop experiences a reduction in growth and yield after 2–3 days of waterlogging, which frequently occurs when they are grown on soils that are poorly drained (Ucan et al., 2007). Wang et al. (2012c) found 13,307 differentially expressed genes (DEGs) in sesame under waterlogging stress. In a more comprehensive study, a total of 1,379 genes were found as the core gene that functions in response to waterlogging. News worthily, they reported 66 genes that may be candidate for improving sesame tolerance to waterlogging (Wang et al., 2016b). Meanwhile, 6 QTLs (*qEZ09ZCL13*, *qWH09CHL15*, *qEZ10ZCL07*, *qWH10ZCL09*, *qEZ10CHL07*, and *qWH10CHL09*) linked to waterlogging traits were identified and a SSR marker (*ZM428*) closely linked to *qWH10CHL09* was further reported as effective marker for marker-assisted selection (MAS) toward waterlogging tolerance (Zhang et al., 2014). Currently, studies are being implemented to unveil genomic variants associated with waterlogging tolerance in sesame.

Concerning drought stress in sesame, few molecular researches have been conducted so far in this field. Using a comparative genomic approach, Dossa et al. (2016b) identified in the whole sesame genome a set of 75 candidate genes for drought tolerance enriched in transcription factors (TFs). Hence, they afterward dissected two important TF families (AP2/ERF and HSF) and proposed some candidate TFs for drought tolerance improvement in sesame (Dossa et al., 2016c,d). Evolutionary analyses of these families showed that sesame has retained most of its drought-related genes similarly as uncovered for its oil-related genes. This may thus explain the relative high drought tolerance observed in this crop. A recent RNA-seq analysis demonstrated that 722 genes act as the core gene set involved in drought responses and 61 candidate genes conferring higher drought tolerance were discovered (Dossa et al., 2017). In another very recent report, an Osmotin-like gene (*SindOLP*) has been uncovered to enhance tolerance to drought, salinity, oxidative stresses, and the charcoal rot pathogen in transgenic sesame (Chowdhury et al., 2017). Finally, a study is underway to decipher SNP variants significantly linked to various drought tolerance traits through an inclusive GWAS.

#### Productivity Enhancement

Although sesame has been domesticated since long time, the yield in most of the growing areas is still very low, thus, hampering its adoption and expansion in the world. Grain yield of sesame per plant is considered to be composed of three components, i.e., the number of capsules per plant, the number of grains per capsule and the grain weight. Some other factors, including plant height, length of capsules, number of capsules per axil and axis height of the first capsule were found to be strongly associated with grain yield of sesame (Biabani and Pakniyat, 2008). Concerning the plant height trait, some QTLs have been reported including *Qph-6* and *Qph-12* (Wu et al., 2014a); 41 QTLs were further identified and the major QTL *qPH-3.3*, was predicted to be responsible for the semi-dwarf sesame plant phenotype (Wang et al., 2016a). However, it contains 102 candidate genes and thus needs further excavation to pinpoint the causative gene. A semi-dwarf gene [*SiGA20ox1* (*SIN_1002659*)] has been recently detected through fine-mapping strategy (Wei et al., 2016). Moreover, two important candidate genes for plant height *SiDFL1* (*SIN_1014512*) and *SiILR1* (*SIN_1018135*) were found in works of Wei et al. (2015). These findings will undoubtedly assist in efforts to create mechanized cultivation varieties with super high yield.

Quantitative trait loci were also identified for the capsule related trait including capsule number per plant (*Qcn-11*), First capsule height (*Qfch-4*, *Qfch-11*, and *Qfch-12*), capsule axis length (*Qcal-5* and *Qcal-9*), capsule length (*Qcl-3*, *Qcl-4*, *Qcl-7*, *Qcl-8*, and *Qcl-12*) (Wu et al., 2014a). Similarly, the gene *SiACS* (*SIN_1006338*) coding for the number of capsule per axil was discovered and may be an important asset for yield improvement in sesame (Wei et al., 2015). Since the number of capsules per axil is related to the number of flowers per axil, Mei et al. (2017) successfully mapped a gene *SiFA* (mono-flower vs. triple-flower) onto the LG11 flanked by the markers *Marker58311* and *Marker36337*.

In regard to the grain yield, few QTLs are actually available including *Qgn-1*, *Qgn-6*, and *Qgn-12* for grain number per capsule and *Qtgw-11* for thousand grain weight.

Flowering time is also an important trait for adaptation of crops to different agro-climatic conditions that significantly affects the yield. Two candidate genes at flowering-time loci *SiDOG1* (*SIN_1022538*) and *SiIAA14* (*SIN_1021838*) have been discovered (Wei et al., 2015). Sesame’s indeterminate growth habit is one of the reasons of its low yielding capacity compared to other oilseed crops (Yol and Uzun, 2012). Recently, the gene *SiDt* (*DS899s00170.023*) was detected as a target gene for conferring the determinate trait in sesame cultivar (Zhang et al., 2016). Also, the branching habit which is an important trait in sesame as it plays a cardinal role in grain yield, cultivation practices and mechanized harvest, has been investigated. A gene *SiBH* controlling the branching habit (uniculm vs. branched type) was mapped onto the LG5 flanked by the markers *Marker129539* and *Marker31462*. This QTL region will be useful for developing unbranched sesame varieties fit for mechanized harvest (Mei et al., 2017).

In another realm, AFLP markers *P01MC08*, *P06MG04* and *P12EA14* were found to be linked to the recessive GMS gene *SiMs1* (Zhao et al., 2013) and 13 SSRs including *SBM298* and *GB50* were associated to the dominant GMS gene *Ms* (Liu et al., 2015). These markers will be valuable for marker-aided breeding of GMS hybrids and harnessing heterosis as one of the promising approaches for yield improvement in sesame (Murty, 1975).

## Current Hot-Topics in Sesame Research and Future Directions

Recent years have witnessed a continuously increasing number of functional genes discovered for key agronomic traits in sesame thanks to the availability of versatile “Omics” tools. In this regard, the next logical step in the sesame research is the functional validation of these gene resources through genetic engineering approaches. Genetic transformation would be an ideal opportunity to quickly transfer the functional genes into sesame elite cultivars. Actually, several successful attempts of sesame genetic transformation through *Agrobacterium* have led to up 42.66% of transformation efficiency (Yadav et al., 2010; Al-Shafeay et al., 2011; Chowdhury et al., 2014). However, improvements of the sesame genetic transformation protocol for reaching higher efficiency are imperative. Nonetheless, studies are in the offing to transfer candidate genes for oil quality traits as well as abiotic stress tolerance into elite cultivars and unveil their molecular mechanisms. In this vein, the first study of the functional analysis in transgenic sesame came into being very recently (Chowdhury et al., 2017). This report presages a bright future for the genetic engineering era of sesame.

Alternatively, the available “Omics” tools actually spur us to enquire scientific questions that remain unexplored or weakly investigated in sesame and could considerably aid in efforts toward its enhancement. These issues constitute the present hot-topics in the sesame research and involve various domains of the crop:

(a) **Origin and domestication of sesame**: Further investigations into the evolution of sesame have been hampered by the absence of detailed molecular data across multiple sesame accessions and wild related species. There is a need for more characterization of wild germplasm including African species, inter-specific crosses and molecular studies to efficiently harness potentialities of sesame wild species. Therefore, the ongoing sequencing projects of wild sesame species and landraces from different origins would help to definitely state the domestication process of sesame.
(b) **Study to solve constraints related to the mechanized harvesting in sesame cultivation:** Plant architecture, dehiscence and indeterminate growth habit are some key factors affecting mechanization of sesame harvest. Many QTLs and genes related to these traits are now available and could be applied through molecular breeding to improve sesame ability for mechanized harvesting.
(c) **Instigation of molecular studies related to root system architecture (RSA), dehiscence character, resistance to disease, tolerance to salt and heat as well as other key agronomic traits for yield improvement:** Sesame root system is thought to play a foremost role in its adaptation to different environments and weather conditions. However, till date, no study has been undertaken to dissect the RSA of sesame and look for some candidate genes linked to this major trait. Furthermore, gene identification for the indehiscence trait in sesame should be a priority in future researches. Also, the available genomic tools need to be more effectively applied for other no less important agronomic traits that could lead to the enhancement of sesame productivity especially the resistance to biotic and abiotic stresses.
(d) **Genetic dissection and exploitation of sesame seed’s bioactive components:** Sesame seeds contain several bioactive components including tocopherols, sesamin, and sesamolin which have tremendous potential for the valorization and value addition of the crop. Hence further studies to investigate the genetic basis of these traits would help to develop nutritionally superior cultivars.
(e) **More “Omics” technologies to support genomic research:** Although several transcriptome data have been released in sesame, future investigations should combine additional “Omics” technologies including proteomics and metabolomics as they are capital for a full understanding of a biological system. Moreover, the available “draft” reference genome sequence of sesame has to be updated to reach a “full sequence” since it may affect the ability to accurately link sequence variations to phenotypes, allele mining or other biological issues.
(f) **Application of advanced biotechnological technologies:** Functional genomics and biotechnological methodologies, such as genetic transformation, yeast assay system, new mutation approach such as Target Induced Local Lesions in Genomes (TILLING) and genome-editing technologies using CRISPR/Cas9 system, are much needed to validate the effects of the available functional genes and their functional variants on the key agronomic traits.

## Conclusion

Sesame has become an emerging crop in the world and its entrance into the “Omics” era has raised it at the “genomic resource-rich crop” level. Invaluable efforts during recent years have engendered several genetic/genomic tools and resources that provide an impetus to research and nurture sesame production for the benefit of smallholder farmers in developing countries. The major traits in sesame including oil and quality, yield related traits, abiotic stress resistance have been thoroughly explored and our understanding of the molecular basis underlying these traits has deeply increased. More importantly, several functional genes, QTLs and molecular markers linked to these traits are now available and could be employed in sesame breeding programs. The current scope in the sesame research concerns the exploitation of these available genomic information for the effective sesame improvement trough molecular- or genomics-assisted breeding. However, the road to raise sesame as one of the major oilseed crops in the world is still long and will need more synergetic efforts, more applications of MAS and inter-disciplinary researches. Other related research fields for the valorization and expansion of sesame should also follow the same trend as the molecular field. Finally and perhaps one of the most important recommendations is to enhance partnerships between national and international sesame teams, so that major issues of sesame production could be addressed through international projects and effective breeding strategies could be implemented.

## Author Contributions

KD, BL, NC, XZ, and DD conceived and designed the paper. KD, LW, XW, YZ, MN, DF, JY, MM, LY, and DD collected and analyzed the literature. KD, LY, MM, and DD drafted the paper. DF, JY, LW, and DD prepared the figures. DF, YZ, LW, XW, MN, DD, XZ, and NC revised the manuscript. All authors have read and approved the final version of the manuscript.

## Conflict of Interest Statement

The authors declare that the research was conducted in the absence of any commercial or financial relationships that could be construed as a potential conflict of interest.

## Footnotes

1 https://www.ncbi.nlm.nih.gov/biosample/SAMN02981519

2 https://www.ncbi.nlm.nih.gov/biosample/SAMN04574066

3 https://www.ncbi.nlm.nih.gov/biosample/SAMN02357081

4 http://ocri-genomics.org/Sinbase

## References

[B1] Abate M. Mekbib F. Ayana A. Nigussie M. (2015). Assessment of genetic diversity in Ethiopian sesame (*Sesamum indicum* L.) germplasm using ISSR markers. *Br. Biotechnol. J.* 8 1–13. 10.9734/BBJ/2015/18481

[B2] Abdellatef E. Sirelkhatem R. Mohamed A. M. M. Radwan K. H. Khalafalla M. M. (2008). Study of genetic diversity in Sudanese sesame (*Sesamum indicum* L.) germplasm using random amplified polymorphic DNA (RAPD) markers. *Afr. J. Biotechnol.* 7 4423–4427.

[B3] Alemu A. Petros Y. Tesfaye K. (2013). Genetic distance of sesame (*Sesamum indicum* L) cultivars and varieties from northwestern Ethiopia using inter simple sequence repeat markers. *East Afr. J. Sci.* 7 31–40. 10.1007/s12298-016-0385-8

[B4] Al-Shafeay A. F. Ibrahim A. S. Nesiem M. R. Tawfik M. S. (2011). Establishment of regeneration and transformation system in Egyptian sesame (*Sesamum indicum* L.) cv Sohag1. *GM Crops* 2 182–192. 10.4161/gmcr.2.3.18378 22179191

[B5] Anilakumar K. R. Pal A. Khanum F. Bawas A. S. (2010). Nutritional, medicinal and industrial uses of sesame (*Sesamum indicum* L.) seeds. *Agric. Conspec. Sci.* 75 159–168.

[B6] Ashri A. (1998). Sesame breeding. *Plant Breed. Rev.* 16 179–228.

[B7] Badri J. Yepuri V. Ghanta A. Siva S. Siddiq E. A. (2014). Development of microsatellite markers in sesame (*Sesamum indicum* L.). *Turk. J. Agric. For.* 38 603–614. 10.3906/tar-1312-104

[B8] Bedigian D. (2003). Evolution of sesame revisited: domestication, diversity and prospects. *Genet. Resour. Crop Evol.* 50 779–787. 10.1023/A:1025029903549

[B9] Bedigian D. (2004). History and lore of sesame in Southwest Asia. *Econ. Bot.* 58 329–353. 10.1663/0013-0001

[B10] Bedigian D. Harlan J. R. (1986). Evidence for cultivation of sesame in the ancient world. *Econ. Bot.* 40 137–154. 10.1007/BF02859136

[B11] Bhat K. V. Babrekar P. P. Lakhanpaul S. (1999). Study of genetic diversity in Indian and exotic sesame (*Sesamum indicum* L.) germplasm using random amplified polymorphic DNA (RAPD) markers. *Euphytica* 110 21–33. 10.1023/A:1003724732323

[B12] Biabani A. R. Pakniyat H. (2008). Evaluation of seed yield-related characters in sesame (*Sesamum indicum* L.) using factor and path analysis. *Pak. J. Biol. Sci.* 11 1157–1160. 10.3923/pjbs.2008.1157.1160 18819557

[B13] Bisht I. S. Mahajan R. K. Loknathan T. R. Agarwal R. C. (1998). Diversity in Indian sesame collection and stratification of germplasm accessions in different diversity groups. *Genet. Resour. Crop Evol.* 45 325–335. 10.1023/A:1008652420477

[B14] Boureima S. Oukarroum A. Diouf M. Cissé N. Van Damme P. (2012). Screening for drought tolerance in mutant germplasm of sesame (*Sesamum indicum*) probing by chlorophyll a fluorescence. *Environ. Exp. Bot.* 81 37–43. 10.1016/j.envexpbot.2012.02.015

[B15] Chen Z. Tonnis B. Morris B. Wang R. R. B. Zhang A. L. Pinnow D. (2014). Variation in seed fatty acid composition and sequence divergence in the FAD2 gene coding region between wild and cultivated sesame. *J. Agri. Food Chem.* 62 11706–11710. 10.1021/jf503648b 25386691

[B16] Cheng F. C. Jinn T. R. Hou R. C. W. Tzen J. T. C. (2006). Neuroprotective effects of sesamin and sesamolin on gerbil brain in cerebral ischemia. *Int. J. Biomed. Sci.* 2 284–288. 23674992 PMC3614603

[B17] Chowdhury S. Basu A. Kundu S. (2014). A new high-frequency *Agrobacterium*-mediated transformation technique for *Sesamum indicum* L. using de-embryonated cotyledon as explant. *Protoplasma* 251 1175–1190. 10.1007/s00709-014-0625-0 24590594

[B18] Chowdhury S. Basu A. Kundu S. (2017). Overexpression of a new osmotin-like protein gene (SindOLP) confers tolerance against biotic and abiotic stresses in sesame. *Front. Plant Sci.* 8:410 10.3389/fpls.2017.00410 PMC5368222 28400780

[B19] Chun J. A. Jin U. H. Lee J. W. Yi Y. B. Hyung N. I. Kang M. H. (2003). Isolation and characterization of a myo-inositol 1-phosphate synthase cDNA from developing sesame (*Sesamum indicum* L.) seeds: functional and differential expression, and salt–induced transcription during germination. *Planta* 216 874–880. 10.1007/s00425-002-0940-0 12624775

[B20] Dixit A. Jin M. H. Chung J. W. Yu J. W. Chung H. K. Ma K. H. (2005). Development of polymorphic microsatellite markers in sesame (*Sesamum indicum* L.). *Mol. Ecol. Notes* 5 736–738. 10.1111/j.1471-8286.2005.01048.x

[B21] Dossa K. (2016). A physical map of important QTLs, functional markers and genes available for sesame breeding programs. *Physiol. Mol. Biol. Plants* 22 613–619. 10.1007/s12298-016-0385-8 27924134 PMC5120042

[B22] Dossa K. Li D. Wang L. Zheng X. Yu J. Wei X. (2017). Dynamic transcriptome landscape of sesame (*Sesamum indicum* L.) under progressive drought and after rewatering. *Genom. Data* 11 122–124. 10.1016/j.gdata.2017.01.003 28180087 PMC5288455

[B23] Dossa K. Diouf D. Cissé N. (2016d). Genome-wide investigation of Hsf genes in sesame reveals their segmental duplication expansion and their active role in drought stress response. *Front. Plant Sci.* 7:1522 10.3389/fpls.2016.01522 PMC5061811 27790233

[B24] Dossa K. Niang M. Assogbadjo A. E. Cisse N. Diouf D. (2016c). Whole genome homology-based identification of candidate genes for drought resistance in (*Sesamum indicum* L.). *Afr. J. Biotechnol.* 15 1464–1475. 10.5897/AJB2016.15420

[B25] Dossa K. Wei X. Li D. Zhang Y. Wang L. Fonceka D. (2016b). Insight into the AP2/ERF transcription factor superfamily in sesame (*Sesamum indicum*) and expression profiling of the DREB subfamily under drought stress. *BMC Plant Biol.* 16:171 10.1186/s12870-016-0859-4 PMC4967514 27475988

[B26] Dossa K. Wei X. Zhang Y. Fonceka D. Yang W. Diouf D. (2016a). Analysis of genetic diversity and population structure of sesame accessions from Africa and Asia as major centers of its cultivation. *Genes* 7:14 10.3390/genes7040014 PMC4846844 27077887

[B27] Feuillet C. Leach E. J. Rogers J. Schnable P. S. Eversole K. (2011). Crop genome sequencing: lessons and rationales. *Trends Plant Sci.* 16 77–88. 10.1016/j.tplants.2010.10.005 21081278

[B28] Food and Agriculture Organization Statistical Databases (FAOSTAT) (2015). *FAOSTAT Provides Free Access to Food and Agriculture Data for Over 245 Countries and Territories and Covers All FAO Regional Groupings.* Available at: http://faostat.fao.org/ [accessed December 19, 2016].

[B29] Frary A. Tekin P. Celik I. Furat S. Uzun B. Doganlar S. (2014). Morphological and molecular diversity in Turkish sesame germplasm and selection of a core set for inclusion in the national collection. *Crop Sci.* 54 1–10. 10.2135/cropsci2012.12.0710

[B30] Fuller D. Q. (2003). Further evidence on the prehistory of sesame. *Asian Agrihist.* 7 127–137.

[B31] Hibasami H. Fujikawa T. Takeda H. Nishibe S. Satoh T. Fujisawa T. (2000). Induction of apoptosis by *Acanthopanax senticosus* HARMS and its component, sesamin in human stomach cancer KATO III cells. *Oncol. Rep.* 7 1213–1216. 11032916 10.3892/or.7.6.1213

[B32] Hiltebrandt V. M. (1932). Sesame (*Sesamum indicum* L.). *Bull. Appl. Bot. Gen. Plant Breed.* 9 1–114.

[B33] Hodgkin T. Brown A. H. D. Hintum T. J. L. V. Morales E. A. V. (1995). *Core collections of Plant Genetic Resources.* London: A co-publication with the International Plant Genetic Resources Institute (IPGRI) and Sayce publishing.

[B34] Huang X. Han B. (2014). Natural variations and genome-wide association studies in crop plants. *Annu. Rev. Plant Biol.* 65 531–551. 10.1146/annurev-arplant-050213-035715 24274033

[B35] IPGRI and NBPGR (2004). *International Crop Network Series 5*. Rome: International Plant Genetic Resources Institute, and *Report of An International Workshop on Okra Genetic Resources*. New Delhi: National Bureau of Indian Plant Genetic Resources, 61.

[B36] Islam F. Gill R. A. Ali B. Farooq M. A. Xu L. Najeeb U. (2016). “Sesame,” in *Breeding Oilseed Crop for Sustainable Production: Opportunities and Constraints*, ed. Gupta S. K. (Cambridge, MA: Academic Press), 135–147.

[B37] Jin U. Lee J. Chung Y. Lee J. Yi Y. Kim Y. (2001). Characterization and temporal expression of a v-6 fatty acid desaturase cDNA from sesame (*Sesamum indicum* L.) seeds. *Plant Sci.* 161 935–941.

[B38] Joshi A. B. (1961). *Sesamum.* Hyderabad: Indian Central Oilseed Committee.

[B39] Kanu P. J. (2011). Biochemical analysis of black and white sesame seeds from China. *Am. J. Biochem. Mol. Biol.* 1 145–157. 10.3923/ajbmb.2011.145.157

[B40] Ke T. Dong C. Han M. Zhao Y. Chen H. Liu H. (2011). Analysis of expression sequence tags from a full-length-enriched cDNA library of developing sesame seeds (*Sesamum indicum*). *BMC Plant Biol.* 11:180 10.1186/1471-2229-11-180 PMC3311628 22195973

[B41] Ke T. Yu J. Dong C. Mao H. Hua W. Liu S. (2015). ocsESTdb: a database of oil crop seed EST sequences for comparative analysis and investigation of a global metabolic network and oil accumulation metabolism. *BMC Plant Biol.* 15:19 10.1186/s12870-014-0399-8 PMC4312456 25604238

[B42] Kim D. H. Zur G. Danin-Poleg Y. Lee S. W. Shim K. B. Kang C. W. (2002). Genetic relationships of sesame germplasm collection as revealed by inter-simple sequence repeats. *Plant Breed.* 121 259–262. 10.1046/j.1439-0523.2002.00700.x

[B43] Kinman M. L. Martin J. A. (1954). Present status of sesame breeding in the United States. *Agron. J.* 46 24–27.

[B44] Kobayashi T. Kinoshita M. Hattori S. Ogawa T. Tsuboi Y. Ishida M. (1990). Development of the sesame metallic fuel performance code. *Nucl. Technol.* 89 183–193.

[B45] Laurentin H. E. Karlovsky P. (2006). Genetic relationship and diversity in a sesame (*Sesamum indicum* L.) germplasm collection using amplified fragment length polymorphism (AFLP). *BMC Genet.* 7:10 10.1186/1471-2156-7-10 PMC1434769 16483380

[B46] Li C. Miao H. Wei L. Zhang T. Han X. Zhang H. (2014). Association mapping of seed oil and protein in *Sesamum indicum* L. using SSR Markers. *PLoS ONE* 9:e105757 10.1371/journal.pone.0105757 PMC4143287 25153139

[B47] Li D. Wang L. Zhang Y. Lv H. Qi X. Wei W. (2012). Pathogenic variation and molecular characterization of *Fusarium* species isolated from wilted sesame in China. *Afr. J. Microbiol. Res.* 6 149–154. 10.5897/AJMR11.1081

[B48] Liu H. M. Tan H. Yu L. Li F. Zhou M. Yang T. (2016). Comparative transcriptome profiling of the fertile and sterile flower buds of a dominant genic male sterile line in sesame (*Sesamum indicum* L.). *BMC Plant Biol.* 16:250 10.1186/s12870-016-0934-x PMC5105256 27832742

[B49] Liu H. Y. Zhou X. A. Wu K. Yang M. M. Zhao Y. Z. (2015). Inheritance and molecular mapping of a novel dominant genic male-sterile gene in *Sesamum indicum* L. *Mol. Breed.* 35:9 10.1007/s11032-015-0189-5

[B50] Mabberley D. J. (1997). *The Plant-Book. A Portable Dictionary of the Higher Plants*, 2nd Edn Cambridge: Cambridge University Press.

[B51] Mei H. Liu Y. Du Z. Wu K. Cui C. Jiang X. (2017). High-density genetic map construction and gene mapping of basal branching habit and flowers per leaf axil in sesame. *Front. Plant Sci.* 8:636 10.3389/fpls.2017.00636 PMC5406510 28496450

[B52] Miyahara Y. Hibasami H. Katsuzaki H. Imai K. Komiya T. (2001). Sesamolin from sesame seed inhibits proliferation by inducing apoptosis in human lymphoid leukemia Molt 4B cells. *Int. J. Mol. Med.* 7 369–371. 10.3892/ijmm.7.4.369 11254875

[B53] Mondal N. Bhat K. V. Srivastava P. S. Sen S. K. (2016). Effects of domestication bottleneck and selection on fatty acid desaturases in Indian sesame germplasm. *Plant Genet. Resour.* 14 81–90. 10.1017/S1479262115000106

[B54] Murty D. S. (1975). Heterosis, combining ability and reciprocal effects for agronomic and chemical characters in Sesamum. *Theor. Appl. Genet.* 45 294–299. 10.1007/BF00276682 24419504

[B55] Nagaraj G. (2009). *Oilseeds: Properties, Processing, Products and Procedures*, 2nd Edn New Delhi: New India Publication.

[B56] Nayar N. M. Mehra K. L. (1970). Sesame. Its uses, botany, World. 2nd Edn. Oxford University Press, Oxford. Cytogenetics and origin. *Econ. Bot.* 24 20–31. 10.1007/BF02860629

[B57] Noguchi T. Ikeda K. Sasaki Y. Yamamoto J. Yamori Y. (2001). Effects of vitamin E and sesamin on hypertension and cerebral thrombogenesis in stroke-prone spontaneously hypertensive rats. *Hypertens. Res.* 24 735–742. 11768736 10.1291/hypres.24.735

[B58] Nyongesa B. O. Were B. A. Gudu S. Dangasuk O. G. Onkware A. O. (2013). Genetic diversity in cultivated sesame (*Sesamum indicum* L.) and related wild species in East Africa. *J. Crop Sci. Biotechnol.* 16 9–15.

[B59] Pandey S. K. Das A. Rai P. Dasgupta T. (2015). Morphological and genetic diversity assessment of sesame (*Sesamum indicum* L.) accessions differing in origin. *Physiol. Mol. Biol. Plants* 21 519–529. 10.1007/s12298-015-0322-2 26600678 PMC4646868

[B60] Park J. Suresh S. Raveendar S. Baek H. Kim C. Lee S. (2015). Development and evaluation of core collection using qualitative and quantitative trait descriptor in sesame (*Sesamum indicum* L.) germplasm. *Korean J. Crop Sci.* 60 75–84.

[B61] Pathak N. Bhaduri A. Bhat K. V. Rai A. K. (2015). Tracking sesamin synthase gene expression through seed maturity in wild and cultivated sesame species – a domestication footprint. *Plant Biol.* 17 1039–1046. 10.1111/plb.12327 25754459

[B62] Pathak N. Rai A. K. Kumari R. Bhat K. V. (2014). Value addition in sesame: a perspective on bioactive components for enhancing utility and profitability. *Pharmacogn. Rev.* 8 147–155. 10.4103/0973-7847.134249 25125886 PMC4127822

[B63] Pazhamala L. Saxena R. K. Singh V. K. Sameerkumar C. V. Kumar V. Sinha P. (2015). Genomics-assisted breeding for boosting crop improvement in pigeonpea (*Cajanus cajan*). *Front. Plant Sci.* 6:50 10.3389/fpls.2015.00050 PMC4330709 25741349

[B64] Pham D. T. Bui M. T. Werlemark G. Bui C. T. Merker A. Carlsson A. S. (2009). A study of genetic diversity of sesame (*Sesamum indicum* L.) in vietnam and cambodia estimated by RAPD markers. *Genet. Resour. Crop. Evol.* 56 679–690. 10.1007/s10722-008-9393-z

[B65] Sankar D. Sambandam G. Ramakrishna R. M. Pugalendi K. V. (2005). Modulation of blood pressure, lipid profiles and redox status in hypertensive patients taking different edible oils. *Clin. Chem. Acta* 355 97–104. 10.1016/j.cccn.2004.12.009 15820483

[B66] Saydut A. Zahir-Duz M. Kaya C. Kafadar A. B. Hamamci C. (2008). Transesterified sesame (*Sesamum indicum* L.) seed oil as a biodiesel fuel. *Bioresour. Technol.* 99 6656–6660. 10.1016/j.biortech.2007.11.063 18178427

[B67] Sehr E. M. Okello-Anyanga W. Hasel-Hohl K. Burg A. Gaubitzer S. Rubaihayo P. R. (2016). Assessment of genetic diversity amongst Ugandan sesame (*Sesamum indicum* L.) landraces based on agromorphological traits and genetic markers. *J. Crop Sci. Biotechnol.* 19 117–129. 10.1007/s12892-015-0105-x

[B68] Spandana B. Reddy V. P. Prasanna G. J. Anuradha G. Sivaramak-rishnan S. (2012). Development and characterization of microsatellite markers (SSR) in Sesamum (*Sesamum indicum* L.) species. *Appl. Biochem. Biotechnol* 168 1594–1607. 10.1007/s12010-012-9881-7 22971833

[B69] Suh M. C. Kim M. J. Hur C. G. Bae J. M. Park Y. I. Chung C. H. (2003). Comparative analysis of expressed sequence tags from *Sesamum indicum* and *Arabidopsis thaliana* developing seeds. *Plant Mol. Biol.* 52 1107–1123. 10.1023/B:PLAN.0000004304.22770.e9 14682612

[B70] Surapaneni M. Yepuri V. Vemireddy L. R. Ghanta A. Siddiq E. A. (2014). Development and characterization of microsatellite markers in Indian sesame (*Sesamum indicum* L.). *Mol. Breed.* 34 1185–1200. 10.1007/s11032-014-0109-0

[B71] Thomson M. J. Septiningsih E. M. Suwardjo F. Santoso T. J. Silitonga T. S. McCouch S. R. (2007). Genetic diversity analysis of traditional and improved Indonesian rice (*Oryza sativa* L.) germplasm using microsatellite markers. *Theor. Appl. Genet.* 114 559–568. 10.1007/s00122-006-0457-1 17136372

[B72] Tiwari S. Kumar S. Gontia I. (2011). Biotechnological approaches for sesame (*Sesamum indicum* L.) and Niger (*Guizotia abyssinica* L.f. Cass.). *As. Pac. J. Mol. Biol. Biotechnol.* 19 2–9.

[B73] Ucan K. Killi F. Gencoglan C. Merdun H. (2007). Effect of irrigation frequency and amount on water use efficiency and yield of sesame (*Sesamum indicum* L.) under field conditions. *Field Crops Res.* 101 249–258.

[B74] Uncu A. O. Frary A. Karlovsky P. Doganlar S. (2016). High-throughput single nucleotide polymorphism (SNP) identification and mapping in the sesame (*Sesamum indicum* L.) genome with genotyping by sequencing (GBS) analysis. *Mol. Breed.* 36:173 10.1007/s11032-016-0604-6

[B75] Uncu A. O. Gultekin V. Allmer J. Frary A. Doganlar S. (2015). Genomic simple sequence repeat markers reveal patterns of genetic relatedness and diversity in sesame. *Plant Genome* 8 1–12. 10.3835/plantgenome2014.11.0087 33228311

[B76] Uzun B. Lee D. Donini P. Cagirgan M. I. (2003). Identification of a molecular marker linked to the closed capsule mutant trait in sesame using AFLP. *Plant Breed.* 122 95–97. 10.1046/j.1439-0523.2003.00787.x

[B77] Varshney R. K. Glaszmann J.-C. Leung H. Ribaut J. M. (2010). More genomic resources for less-studied crops. *Trends Biotechnol.* 28 452–460. 10.1016/j.tibtech.2010.06.007 20692061

[B78] Varshney R. K. Nayak S. N. May G. D. Jackson S. A. (2009). Next-generation sequencing technologies and their implications for crop genetics and breeding. *Trends Biotechnol.* 27 522–530. 10.1016/j.tibtech.2009.05.006 19679362

[B79] Verma P. Goyal R. Chahota R. K. Sharma T. R. Abdin M. Z. Bhatia S. (2015). Construction of a genetic linkage map and identification of QTLs for seed weight and seed size traits in Lentil (Lens culinaris Medik.). *PLoS ONE* 10:e0139666 10.1371/journal.pone.0139666 PMC4593543 26436554

[B80] Wang L. Han X. Zhang Y. Li D. Wei X. Ding X. (2014c). Deep resequencing reveals allelic variation in *Sesamum indicum*. *BMC Plant Biol.* 14:225 10.1186/s12870-014-0225-3 PMC4148021 25138716

[B81] Wang L. Li D. Zhang Y. Gao Y. Yu J. Wei X. (2016b). Tolerant and susceptible sesame genotypes reveal waterlogging stress response patterns. *PLoS ONE* 11:e0149912 10.1371/journal.pone.0149912 PMC4774966 26934874

[B82] Wang L. Xia Q. Zhang Y. Zhu X. Zhu X. Li D. (2016a). Updated sesame genome assembly and fine mapping of plant height and seed coat color QTLs using a new high-density genetic map. *BMC Genome* 17:31 10.1186/s12864-015-2316-4 PMC4702397 26732604

[B83] Wang L. Yu J. Li D. Zhang X. (2014b). Sinbase: an integrated database to study genomics, genetics and comparative genomics in *Sesamum indicum*. *Plant Cell Physiol.* 56:e2 10.1093/pcp/pcu175 25480115

[B84] Wang L. Yu S. Tong C. Zhao Y. Liu Y. Song C. (2014a). Genome sequencing of the high oil crop sesame. *Genome Biol.* 15:R39 10.1186/gb-2014-15-2-r39 PMC4053841 24576357

[B85] Wang L. Zhang Y. Li P. Wang X. Zhang W. Wei W. (2012a). HPLC analysis of seed sesamin and sesamolin variation in a sesame germplasm collection in China. *J. Am. Oil Chem. Soc.* 8 1011–1020. 10.1007/s11746-011-2005-7

[B86] Wang L. Zhang Y. Qi X. Gao Y. Zhang X. (2012b). Development and characterization of 59 polymorphic cDNA-SSR markers for the edible oil crop *Sesamum indicum* (Pedaliaceae). *Am. J. Bot.* 99 394–398. 10.3732/ajb.1200081 23002163

[B87] Wang L. Zhang Y. Qi X. Li D. Wei W. Zhang X. (2012c). Global gene expression responses to waterlogging in roots of sesame (*Sesamum indicum* L.). *Acta Physiol. Plant.* 34 2241–2249. 10.1007/s11738-012-1024-9

[B88] Wei L. Miao H. Li C. Duan Y. Niu J. Zhang T. (2014). Development of SNP and InDel markers via de novo transcriptome assembly in *Sesamum indicum* L. *Mol. Breed.* 34 2205–2217. 10.1007/s11032-014-0174-4

[B89] Wei L. Miao H. Zhang H. (2012). Transcriptomic analysis of sesame development. *Sci. Agric. Sin.* 45 1246–1256. 10.3864/j.issn.0578-1752.2012.07.002

[B90] Wei L. Zhang H. Zheng Y. Guo W. Zhang T. (2008). Development and utilization of EST-derived microsatellites in sesame (*Sesamum indicum* L.). *Acta Agron. Sin.* 34 2077–2084.

[B91] Wei L. Zhang H. Zheng Y. Miao H. Zhang T. Guo W. (2009). A genetic linkage map construction for sesame (*Sesamum indicum* L.). *Genes Genomics* 31 199–208. 10.1007/BF03191152

[B92] Wei W. Qi X. Wang L. Zhang Y. Hua W. Li D. (2011). Characterization of the sesame (*Sesamum indicum* L.) global transcriptome using Illumina paired end sequencing and development of EST SSR markers. *BMC Genomics* 12:451 10.1186/1471-2164-12-451 PMC3184296 21929789

[B93] Wei W. Zhang Y. Lv H. Li D. Wang L. Zhang X. (2013). Association analysis for quality traits in a diverse panel of Chinese sesame (*Sesamum indicum* L.) *germplasm*. *J. Integr. Plant Biol.* 55 745–758. 10.1111/jipb.1204 23570323

[B94] Wei X. Gong H. Yu J. Liu P. Wang L. Zhang Y. (2017). Sesame FG: an integrated database for the functional genomics of sesame. *Sci. Rep.* 7:2342 10.1038/s41598-017-02586-3 PMC5443765 28539606

[B95] Wei X. Liu K. Zhang Y. Feng Q. Wang L. Zhao Y. (2015). Genetic discovery for oil production and quality in sesame. *Nat. Commun.* 6 8609 10.1038/ncomms9609 PMC4634326 26477832

[B96] Wei X. Wang L. Zhang Y. Qi X. Wang X. Ding X. (2014). Development of simple sequence repeat (SSR) markers of sesame (*Sesamum indicum*) from a genome survey. *Molecules* 19 5150–5162. 10.3390/molecules19045150 24759074 PMC6270694

[B97] Wei X. Zhu X. Yu J. Wang L. Zhang Y. Li D. (2016). Identification of sesame genomic variations from genome comparison of landrace and variety. *Front. Plant Sci.* 7:1169 10.3389/fpls.2016.01169 PMC4971434 27536315

[B98] Weiss E. A. (2000). *Sesame. Oilseed Crops*, 2nd Edn London: Blackwell Science.

[B99] Witcombe J. R. Hollington P. A. Howarth C. J. Reader S. Steele K. A. (2007). Breeding for abiotic stresses for sustainable agriculture. *Philos. Trans. R. Soc. B.* 363 703–716. 10.1098/rstb.2007.2179 PMC2610105 17761467

[B100] Wongyai W. Saengkaewsook W. Veerawudh J. (2001). “Sesame mutation induction: improvement of non-shattering capsule by using gamma rays and EMS,” in *Sesame Improvement by Induced Mutations, IAEA-TECDOC-1195*, ed. van Zanten L. (Vienna: IAEA), 71–78.

[B101] Wu K. Liu H. Yang M. Tao Y. Ma H. Wu W. (2014a). High-density genetic map construction and QTLs analysis of grain yield-related traits in sesame (*Sesamum indicum* L.) based on RAD-Seq technology. *BMC Plant Biol.* 14:274 10.1186/s12870-014-0274-7 PMC4200128 25300176

[B102] Wu K. Yang M. Liu H. Tao Y. Mei J. Zhao Y. (2014b). Genetic analysis and molecular characterization of Chinese sesame (*Sesamum indicum* L.) cultivars using Insertion-Deletion (InDel) and Simple Sequence Repeat (SSR) markers. *BMC Genet.* 15:35 10.1186/1471-2156-15-35 PMC4234512 24641723

[B103] Yadav M. Sainger D. C. M. Jaiwal P. K. (2010). *Agrobacterium tumefaciens*-mediated genetic transformation of sesame (*Sesamum indicum* L.). *Plant Cell Tissue Organ Cult* 103 377–386. 10.1007/s11240-010-9791-8

[B104] Yi D. K. Kim K. J. (2012). Complete chloroplast genome sequences of important oilseed crop *Sesamum indicum* L. *PLoS ONE* 7:e35872 10.1371/journal.pone.0035872 PMC3351433 22606240

[B105] Yol E. Uzun B. (2012). Geographical patterns of sesame (*Sesamum indicum* L.) accessions grown under Mediterranean environmental conditions, and establishment of a core collection. *Crop Sci.* 52 2206–2214. 10.2135/cropsci2011.07.0355

[B106] Yu J. Dossa K. Wang L. Zhang Y. Wei X. Liao B. (2016). PMDBase: a database for studying microsatellite DNA and marker development in plants. *Nucleic Acids Res.* 45 D1046–D1053. 10.1093/nar/gkw906 27733507 PMC5210622

[B107] Yu J. Ke T. Tehrim S. Sun F. Liao B. Hua W. (2015). PTGBase: an integrated database to study tandem duplicated genes in plants. *Database (Oxford)* 2015:bav017 10.1093/database/bav017 PMC4369376 25797062

[B108] Yu J. Wang L. Guo H. Liao B. King G. Zhang X. (2017). Genome evolutionary dynamics followed by diversifying selection explains the complexity of the *Sesamum indicum* genome. *BMC Genomics* 18:257 10.1186/s12864-017-3599-4 PMC5364699 28340563

[B109] Zamir D. (2001). Improving plant breeding with exotic genetic libraries. *Nat. Rev. Genet.* 2 983–989. 10.1038/35103590 11733751

[B110] Zeven A. Zhukovsky P. (1975). *Dictionary of Cultivated Plants and Their Centres of Diversity.* Wageningen: PUDOC.

[B111] Zhang H. Miao H. Li C. Wei L. Duan Y. Ma Q. (2016). Ultra-dense SNP genetic map construction and identification of SiDt gene controlling the determinate growth habit in *Sesamum indicum* L. *Sci. Rep.* 6:31556 10.1038/srep31556 PMC4985745 27527492

[B112] Zhang H. Li C. Miao H. Xiong S. (2013b). Insights from the complete chloroplast genome into the evolution of *Sesamum indicum* L. *PLoS ONE* 8:e80508 10.1371/journal.pone.0080508 PMC3841184 24303020

[B113] Zhang H. Miao H. Wang L. Qu L. Liu H. Wang Q. (2013a). Genome sequencing of the important oilseed crop *Sesamum indicum* L. *Genome Biol.* 14:401 10.1186/gb-2013-14-1-401 PMC3663098 23369264

[B114] Zhang H. Miao H. Wei L. Li C. Zhao R. Wang C. (2013c). Genetic analysis and QTL mapping of seed coat color in sesame (*Sesamum indicum* L.). *PLoS ONE* 8:e63898 10.1371/journal.pone.0063898 PMC3660586 23704951

[B115] Zhang H. Wei L. Miao H. Zhang T. Wang C. (2012). Development and validation of genic-SSR markers in sesame by RNA-seq. *BMC Genomics* 13:316 10.1186/1471-2164-13-316 PMC3428654 22800194

[B116] Zhang X. Zhao Y. Cheng Y. Feng X. Guo Q. Zhou M. (2000). Establishment of sesame germplasm core collection in China. *Genet. Resour. Crop Evol.* 47 273–279. 10.1023/A:1008767307675

[B117] Zhang Y. Wang L. Li D. Gao Y. Lu H. Zhang X. (2014). Mapping of sesame waterlogging tolerance QTL and identification of excellent waterlogging tolerant germplasm. *Sci. Agric. Sin.* 47 422–430.

[B118] Zhang Y. Wang L. Xin H. Li D. Ma C. Ding X. (2013). Construction of a high-density genetic map for sesame based on large scale marker development by specific length amplified fragment (SLAF) sequencing. *BMC Plant Biol.* 13:141 10.1186/1471-2229-13-141 PMC3852768 24060091

[B119] Zhang Y. Zhang X. Che Z. Wang L. Wei W. Li D. (2012). Genetic diversity assessment of sesame core collection in China by phenotype and molecular markers and extraction of a mini-core collection. *BMC Genet.* 13:102 10.1186/1471-2156-13-102 PMC3574832 23153260

[B120] Zhao Y. Yang M. Wu K. Liu H. Wu J. Liu K. (2013). Characterization and genetic mapping of a novel recessive genic male sterile gene in sesame (*Sesamum indicum* L.). *Mol. Breed.* 32 901–908. 10.1007/s11032-013-9919-8

